# Macular Microvascular Perfusion Status in Hypertensive Patients with Chronic Kidney Disease

**DOI:** 10.3390/jcm12175493

**Published:** 2023-08-24

**Authors:** Heiko Stino, Elisa de Llano Pato, Irene Steiner, Nikolaus Mahnert, Maximilian Pawloff, Matthias Hasun, Franz Weidinger, Ursula Schmidt-Erfurth, Andreas Pollreisz

**Affiliations:** 1 Department of Ophthalmology, Medical University of Vienna, 1090 Vienna, Austria; 2 Vienna Reading Center, Medical University of Vienna, 1090 Vienna, Austria; 3 Center for Medical Data Science, Institute of Medical Statistics, Medical University of Vienna, 1090 Vienna, Austria; 4 Department of Cardiology, Clinic Land Strasse, Vienna Healthcare Group, 1030 Vienna, Austria; matthias.hasun@wienkav.at (M.H.); franz.weidinger@wienkav.at (F.W.)

**Keywords:** retina, macula, chronic kidney disease, hypertension, optical coherence tomography angiography, vessel density, fractal dimension, perfusion

## Abstract

To compare retinal microvascular perfusion between the eyes of hypertensive patients with and without chronic kidney disease (CKD), the vessel density (VD) and fractal dimension (FD) of the superficial (SVP) and deep retinal vascular plexus (DVP) were analyzed on 6 × 6 mm fovea-centered optical coherence tomography angiography (OCTA) images of patients with hypertension. The retina was divided into an inner ring (IR) and outer ring (OR) according to the Early Treatment of Diabetic Retinopathy Study grid. The glomerular filtration rate (GFR) was determined and CKD was diagnosed (GFR < 60 mL/min/1.73 m^2^). Ninety-six eyes from 52 patients with hypertension were included in this analysis. Twenty patients (*n* = 37 eyes) were diagnosed with CKD. The mean age was 69 ± 11.7 years and 60.4 ± 9.2 years in the CKD group and in the control group, respectively. The univariate model revealed a significant difference in VD between patients without and with CKD in the superficial IR (0.36 ± 0.03 vs. 0.34 ± 0.04, *p* = 0.03), the superficial OR (0.35 ± 0.02 vs. 0.33 ± 0.04, *p* = 0.02), the deep OR (0.24 ± 0.01 vs. 0.23 ± 0.02, *p* = 0.003), and the FD in the SVP (1.87 ± 0.01 vs. 1.86 ± 0.02, *p* = 0.02) and DVP (1.83 ± 0.01 vs. 1.82 ± 0.01, *p* = 0.006). After adjusting for age and sex, these differences did not remain statistically significant. Similar results were observed for the FD in the SVP and DVP. In our cohort, patients with hypertension and CKD did not differ from patients without CKD in regard to microvascular perfusion status in the macular area as assessed using OCTA.

## 1. Introduction

Chronic kidney disease (CKD) is a worldwide public health concern with a rising incidence and prevalence. Although adverse outcomes can be prevented or delayed through early treatment, CKD remains underdiagnosed und undertreated [1].

It has been shown that the development and progression of CKD are associated with loss of the microvasculature [2,3]. To evaluate kidney function, the glomerular filtration rate (GFR) expressed on a continuous scale is still the standard, with most publications defining CKD as a GFR of less than 60 mL/min/1.73 m^2^ [1,3,4,5,6,7,8,9]. Since direct visualization of the kidney’s microvascular structure without invasive methods is impossible, retinal vessels reflecting changes in systemic microcirculation have been discussed for microvascular analysis [10,11]. Hypertension is not only a main cause of CKD but is also associated with faster disease progression and cardiovascular disease [1,2]. Sabanayagam et al., found that microvascular changes in the retina increased the risk for CKD in hypertensive patients, suggesting that retinal image analysis may aid in risk assessment [9].

Various studies have investigated the relationship between vascular diameters and risk factors for cardiovascular diseases [12,13,14], diabetes [15], and CKD [5,8,9,16] from fundus images.

A recently introduced deep learning algorithm showed good performance in detecting CKD on mydriatic color fundus photographs, highlighting the importance of retinal imaging as a non-invasive screening tool [6].

Another potentially important method in screening for changes in retinal microvasculature is the analysis of fractal dimension (FD) as a global measure for vascular complexity expressed as a single variable, mostly evaluated from fundus photographs [4,7,16,17]. With the introduction of optical coherence tomography angiography (OCTA), high-resolution angiograms and analysis of the vessel density (VD) have become additional microvascular parameters associated with the monitoring of various systemic diseases, including hypertension and impaired kidney function [18,19,20].

Hypertension and CKD are closely related in that they both cause changes in the microvasculature of the neuroretina, which can be detected with various imaging methods.

The aim of this study was to evaluate changes associated with CKD in patients with hypertension by analyzing retinal microvascular parameters on OCTA en-face images in the superficial (SVP) and deep vascular plexus (DVP).

## 2. Materials and Methods

In this cross-sectional study, patients with hypertension were recruited from the Clinic Landstrasse cardiology department (Vienna, Austria). The study was conducted according to the guidelines of the Declaration of Helsinki. Approval was given from the Medical University of Vienna Ethics Committee. Written informed consent was obtained from all subjects prior to enrollment in the study. People with diabetes and other ocular diseases were excluded from this analysis.

GFR was calculated from serum creatinine concentration using the CKD Epidemiology Collaboration equation [21]. Patients were divided into groups depending on their GFR level, as described in the K/DOQI clinical practice guidelines of the National Kidney Foundation (1): the cut-off for diagnosis of CKD was a GFR of less than 60 mL/min/1.73 m^2^ for a period of more than 3 months. GFR levels of ≥90 mL/min/1.73 m^2^, 60–89 mL/min/1.73 m^2^, 30–59 mL/min/1.73 m^2^, 15–29 mL/min/1.73 m^2^, and <15 mL/min/1.73 m^2^ were further defined as no CKD, mild, moderate, severe CKD, and kidney failure, respectively.

Fovea-centered 6 × 6 mm optical coherence tomography angiography (OCTA) scans were acquired using a 200 kHz Plex Elite 9000 device (Carl Zeiss Meditec Inc., Dublin, CA, USA). Automated segmented SVP and DVP en-face images were exported for further analysis of microvascular parameters.

**Figure 1:**
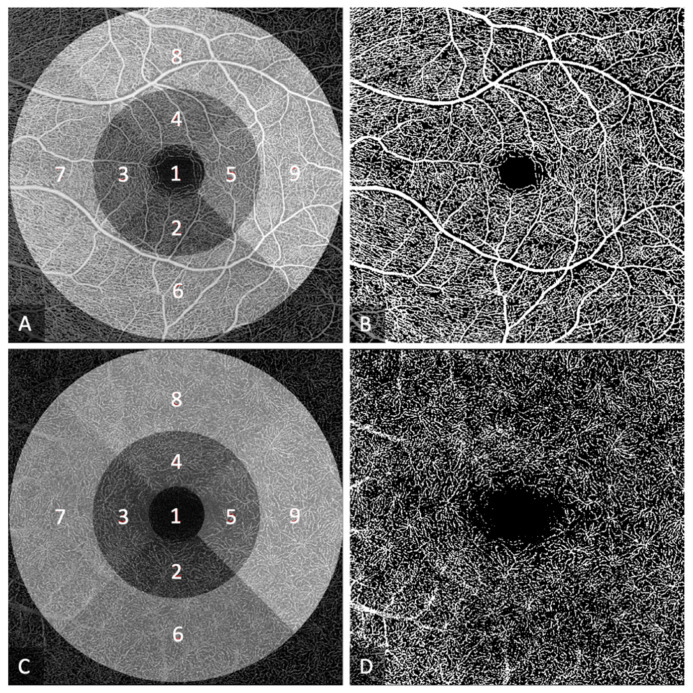
Calculation of vessel density (VD) in the superficial vascular plexus (SVP) (**A**,**B**) and deep vascular plexus (DVP) (**C**,**D**). Images (**A**,**C**) show the grid used for analysis: central field (1); inner ring (2–5) consisting of inner inferior (2), inner temporal (3), inner superior (4), and inner nasal (5) fields; outer ring (6–9) consisting of outer inferior (6), outer temporal (7), outer superior (8), and outer nasal (9) fields. Images (**B**,**D**) show the binarized images of the SVP and DVP before exclusion of the big vessels.

We calculated the VD as the percentage of vessel area with blood flow over the total area measured (Figure 1) and used the software Fractalyse to calculate the FD with the box-counting method, as most commonly reported in the literature [22]. Both calculations were performed on binary images that segmented the vessels. For superficial images, a predefined threshold was used, while for deep images, the optimal threshold was calculated based on the individual image properties.

To evaluate the microcirculation of the macula, larger vessels were excluded from the VD calculation. VD was calculated in all individual sectors of the 6 mm ETDRS grid centered around the fovea. To place the grid, the center of the foveal avascular zone (FAZ) was manually annotated on the en-face OCTA image using ImageJ (National Institutes of Health). An automated algorithm developed by the Vienna Reading Center at the Medical University of Vienna was used to perform the VD calculations. The FAZ was measured on en-face images of the SVP.

### Statistical Analyses

Qualitative variables are reported as absolute frequencies and percentages. Metric variables are summarized as mean ± standard deviation (SD). Univariate mixed models (SAS Proc mixed) were calculated with the patient as a random factor to consider that some patients have measurements on both eyes. The number of degrees of freedom was calculated using Kenward–Roger approximation. The dependent variables were SVP VD in the inner ring (=mean of inner superior, IS; inner temporal, IT; inner nasal, IN; inner inferior, II) and the outer ring (=mean of outer superior, OS; outer temporal, OT; outer nasal, ON; outer inferior, OI), DVP VD in the inner ring (=mean of IS, IT, IN, II) and the outer ring (=mean of OS, OT, ON, OI), SVP FD, DVP FD, and FAZ size on the SVP. The independent variable was CKD (dichotomous). If the *p*-value was <0.05, a multivariable mixed model was applied with age and sex as additional independent variables. The mixed models were also calculated for each subarea (superior, temporal, nasal, inferior) in the superior and deep plexus separately, whereby Bonferroni correction was applied to adjust for the number of subareas (4 subareas). Analyses were repeated with GFR (metric) instead of CKD as the independent variable. The effect of age on GFR was analyzed using a linear regression model. Statistical analyses were carried out with SAS 9.4. and R 4.1.3. The significance level was set to alpha = 0.05. Due to the exploratory character of the analyses, multiplicity correction was performed for the number of subareas, but not for the number of endpoints. Hence, the interpretation of the *p*-values is descriptive.

## 3. Results

**Figure 2:**
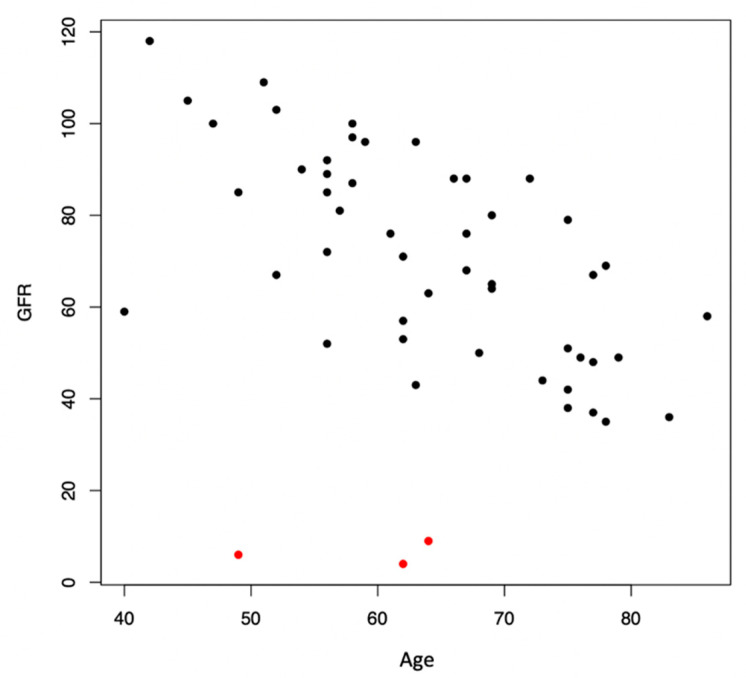
Scatter plot of glomerular filtration rate (GFR) and patient age. Age was significantly negatively associated with GFR (*p* = 0.0007). Three patients with end-stage CKD (GFR < 15 mL/min/1.73 m^2^) are highlighted in red.

Ninety-six eyes (49% right and 51% left eyes) of 52 patients (50% female) were included in this cross-sectional study. The mean age of all patients was 64 ± 10.9 years (range 40–86 years). Twenty patients (37 eyes) had a GFR of less than 60 mL/min/1.73 m^2^ and were therefore classified as having CKD. Thirty-two patients (59 eyes) had a GFR of ≥60 mL/min/1.73 m^2^ and thus composed the control group. The mean age was 69 ± 11.7 years (range 40–86 years) and 60.4 ± 9.2 years (range 42–78 years) in the CKD group and in the control group, respectively. The mean GFR was 41 ± 16.6 mL/min/1.73 m^2^ and 84.8 ± 14.4 mL/min/1.73 m^2^ in the CKD group and the control group, respectively. Age was significantly negatively associated with GFR (estimate −1.1 [95%CI–1.71; −0.49] *p* = 0.0007), as shown in Figure 2.

Detailed reports of VD, FD, and FAZ are specified in Table 1.

After assessment of GFR levels, no, mild, and moderate CKD and end-stage/kidney failure were determined for 11 (21%), 21 (40%), 17 (33%), and 3 patients (6%), respectively. However, no further analyses were performed using these subgroups due to the small sample sizes.

### 3.1. Vessel Density

The mean SVP VD (CKD vs. control) was 0.34 ± 0.04 vs. 0.36 ± 0.03 in the inner ring and 0.33 ± 0.04 vs. 0.35 ± 0.02 in the outer ring. In the univariate mixed model, we found significantly lower VDs in patients with CKD in the inner ring (estimate [95% CI]: −0.021 [−0.039; −0.002], *p* = 0.033) and outer ring (−0.019 [−0.035; −0.004], *p* = 0.017). However, after adjusting for age and sex, these differences did not remain statistically significant in either the inner ring (estimate [95% CI]: −0.011 [−0.031; 0.009], *p* = 0. 27) or outer ring (−0.012 [−0.029; 0.004], *p* = 0.14).

The mean DVP VD (CKD vs. control) was 0.23 ± 0.03 vs. 0.24 ± 0.03 in the inner ring and 0.23 ± 0.02 vs. 0.24 ± 0.01 in the outer ring. The univariate mixed model revealed no significant difference in the inner ring (−0.006 [−0.022; 0.009] *p* = 0.40) but a significantly lower VD in patients with CKD in the outer ring (−0.012 [−0.019; −0.004] *p* = 0. 0035). This difference did not remain significant after adjusting for age and sex (−0.004 [−0.011; 0.003] *p* = 0.26)

### 3.2. Fractal Dimension

The FD (CKD vs. control) was 1.86 ± 0.02 vs. 1.87 ± 0.01 in the SVP and 1.82 ± 0.01 vs. 1.83 ± 0.01 in the DVP. The univariate mixed model revealed a significantly lower FD in the SVP (−0.010 [−0.018; −0.0013], *p* = 0. 024) and DVP (−0.006 [−0.010; −0.002], *p* = 0.0065). After adjusting for age and sex, these differences did not remain statistically significant in the SVP (−0.007 [−0.016; 0.002] *p* = 0.14) or DVP (−0.002 [−0.005; 0.002] *p* = 0.38).

### 3.3. Foveal Avascular Zone

The FAZ (CKD vs. controls) was 209.3 ± 73.9 µm^2^ vs. 247 ± 86.8 µm^2^ with a borderline significant effect (−47.1 [−93.7; −0.5] *p* = 0. 048), which did not remain when adjusted for age and sex (−41.2 [−91.3; 8.9] *p* = 0.11).

**Figure 3:**
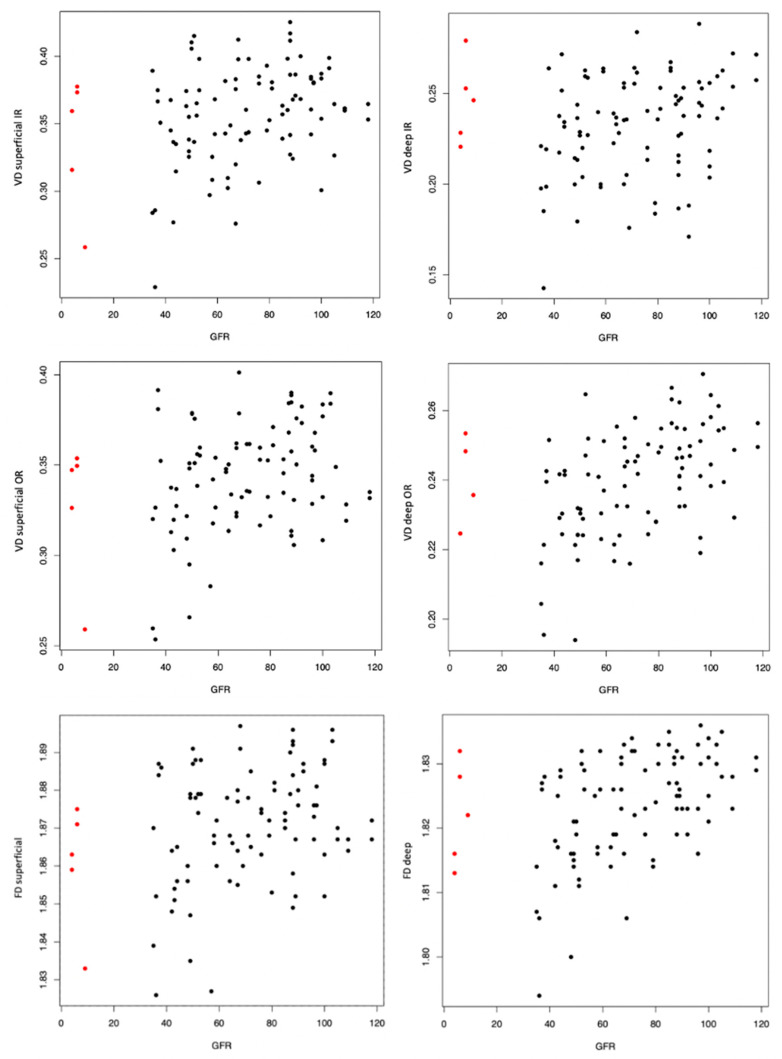
Scatter plots of glomerular filtration rate (GFR) and vessel density (VD) in the superficial and deep inner ring (IR) and outer ring (OR). Scatter plot of GFR and fractal dimension (FD) is specified over the whole retinal area. Except for the deep IR, the univariate model revealed a statistically significant association of all microvascular parameters with GFR (*p* < 0.05), which did not remain significant after adjusting for age and sex. Five eyes from three patients with end-stage CKD (GFR < 15 mL/min/1.73 m^2^) are highlighted in red.

When including GFR instead of CKD in the mixed models, the results for VD and FD were similar, revealing a significant association of GFR with vascular parameters in the univariate model (*p* < 0.05) (as shown in Figure 3), which lost significance after adjusting for age and sex (*p* > 0.05).

## 4. Discussion

In our cohort, hypertensive patients with CKD did not show any differences in retinal microvascular perfusion status in the macular area in comparison to hypertensive patients without CKD.

Studies have shown that patients with hypertension show signs of retinopathy [23] or microvascular changes [24] and are at risk for development or progression of CKD [1,2]. It is therefore important to screen this patient cohort effectively for risk factors predicting CKD. Although reports on certain vascular markers vary, retinal imaging is still a potentially valuable tool for microvascular assessment of systemic diseases [10]. With CKD being associated with retinal microvascular changes such as retinopathy lesions or various ocular diseases, understanding the pathophysiology of these diseases will enable the development of new screening methods [11].

Various authors have evaluated the association of microvascular structure as seen on color fundus images and kidney parameters with different results [3,4,5,9,16,24]. Ooi et al., showed that patients with CKD had a narrowed microvasculature as measured on color fundus images [3]. Sabanayagam et al., reported that retinal arteriolar narrowing in patients with hypertension increased the likelihood of CKD [9]. Lim et al., found that a smaller FD and narrower retinal arteriolar calibers on color fundus images were significantly associated with lower GFR levels, but not, however, with CKD defined as <60 mL/min/1.73 m^2^ [16]. On the contrary, Sabanayagam et al., reported no causal relationship between vessel diameters in CKD in a large cohort of 3199 adults [5]. Grunwald et al., likewise found no relationship between GFR and vessel calibers in 2605 patients. However, they described that vascular abnormalities, which are usually related to hypertension, were associated with lower GFR levels [24]. Wong et al., also showed that retinal microvascular abnormalities are associated with renal dysfunction using common systemic processes in a large, population-based investigation consisting of 10,056 people [25]. Sabanayagam et al., showed that a deep learning algorithm can detect CKD from CF images and therefore be used as an adjunctive or opportunistic screening tool [6].

Further analyses of vascular parameters using different imaging modalities are crucial, as results vary between authors, but evidence suggests that retinal microvascular parameters can reflect renal function.

The introduction of swept-source OCTA, providing quick and non-invasive high-resolution angiograms, enables the assessment of microvascular parameters such as VD and FD in different capillary plexus layers. This allows new insight into pathophysiological processes of various diseases including CKD.

Various authors have evaluated the association of kidney parameters with microvascular changes as seen with OCTA. Wang et al., investigated the association of renal function with the retinal VD of the SVP in patients with type 2 diabetes on 3 × 3 mm fovea-centered OCTA images. They found a significantly lower parafoveal VD in patients with CKD compared to patients without CKD and that the GFR was associated with VD (β = 0.029) [19]. Wu et al., also analyzed 3 × 3 mm fovea-centered angiograms and found that patients with CKD demonstrated a reduction in macular thickness and changes in neuroretinal parameters which correlated with microvascular rarefaction in the SVP. However, they found no direct correlation of CKD or GFR levels with VD [18]. Wang et al., acquired 3 × 3 mm fovea-centered OCTA scans and assessed kidney function in patients with diabetes without diabetic retinopathy. They described that a lower VD in the SVP was associated with a higher urinary albumin/creatinine ratio and a higher prevalence of macroalbuminuria [26]. You et al., discovered in a large population-based study using 6 × 6 mm fovea-centered scans that a lower VD in the DVP was associated with a higher level of creatinine [27].

Peng et al., assessed VD within the SVP and DVP using 6 × 6 fovea-centered OCTA images, revealing that patients with hypertension also exhibited a decreased DVP VD compared to those without hypertension. SVP density was only reduced in patients with signs of hypertensive retinopathy [20]. In contrast, Hua et al., described a reduction in superficial VD in patients with a history of hypertension without hypertensive retinopathy, but no differences in the DVP compared to healthy controls [28]. In another study, they also reported a reduction in the SVP VD in patients with poor blood pressure control in comparison to patients with standard or intensive control [29]. For patients with hypertensive retinopathy, Liu et al., proposed OCTA-based staging, highlighting its potential as a useful tool for screening and monitoring [30].

In our cohort, all patients had hypertension without signs of retinopathy. However, the differences between patients with CKD and the control group observed in the univariate mixed model were not statistically significant after adjusting for age and sex.

In 1990, retinal branching patterns were first characterized as fractals, a geometrical pattern whose parts resemble the whole, providing a new measure of vascular geometry in ocular disease [31]. This allowed a better analyses of retinal anatomy, which led to a variety of heterogenous studies evaluating FD with different imaging modalities and calculation methods [22].

Sng et al., analyzed FD in patients with CKD using two 45° fundus photographs (optic disc and fovea-centered) and showed a reduced FD in patients with CKD compared to patients without CKD (1.43 ± 0.048 vs. 1.44 ± 0.042) [7]. Lim et al., found that patients with smaller FDs on fundus images had higher likelihoods of having microalbuminuria but no significant correlation between FD and CKD (16). Paterson et al., described a negative correlation of arteriolar fractal dimension with CKD stages 4–5 on 45° fundus images [4]. Broe et al., found that a lower FD significantly predicts diabetic nephropathy in diabetic patients [17].

Zahid et al., were among the first to investigate FD on OCTA images in the eyes of patients with diabetic retinopathy without macular edema and found significant reductions in the SVP and DVP [32]. However, these analyses have not been performed in patients with hypertension to assess concomitant CKD.

In our study, we found a reduction in the FD in the SVP (1.86 ± 0.02 vs. 1.87 ± 0.01) and the DVP (1.82 ± 0.01 vs. 1.83 ± 0.01), which did not remain significant after adjusting for age and sex.

Age and sex both have an influence on microvascular parameters in OCTA analysis and have to be accounted for when interpretating data [33,34]. However, age is also an important factor to consider in patients with CKD. A large study investigating reports of over 200,000 people with CKD revealed that almost 50% of the patients were aged 75 years or older [35]. In our cohort, patients with CKD also tended to be older (which was significantly associated with GFR levels). You et al., analyzed macular VD in a large population-based study consisting of 2018 healthy adults. Their results are in line with previous findings as they also detected higher VDs in the SVP and DVP in younger patients. However, they reported stable values until age 50, with a progressive decrease of only 1.3% per decade afterwards. While the DVP is the area in which a high association with creatinine levels has been described, an association with GFR could not be observed in our cohort after adjusting for age and sex [27].

Our cohort was small, consisting of only 96 eyes from 52 patients. However, patients with CKD showed a higher decrease in VD in the SVP than expected due to normal age-differences, as reported in a recent population-based study [27]. Larger cohorts with age-matched controls might be more important in patients with systemic diseases such as CKD to precisely evaluate their influence on microvascular parameters on OCTA images. Due to the high association of GFR with age, it is therefore difficult to determine the influence of each parameter separately in our cohort.

Macular microvascular perfusion in patients with hypertension and CKD did not show significant differences compared to in patients without CKD when assessed using OCTA.

## Figures and Tables

**Table 1:** Demographics of patients, vessel density in the superficial and deep vascular plexus, fractal dimension in the superficial and deep vascular plexus, and foveal avascular zone in the superficial plexus in patients with and without chronic kidney disease (CKD) depending on glomerular filtration rate (GFR). *p*-values are specified for CKD and GFR as independent variables.

Statistics, Mean ± SD	no CKD (GFR > 60 mL/min/1.73 m^2^)	CKD (GFR < 60 mL/min/1.73 m^2^)	*p* Univariate (CKD)	*p* Adjusted (CKD)	*p* Univariate (GFR)	*p* Adjusted (GFR)
*n* of patients (eyes)	32 (59)	20 (37)				
*n* female (%)	17 (53%)	9 (45%)	-	-	-	-
Age (years)	60.4 ± 9.2	69 ± 11.7	-	-	-	-
GFR (mL/min/1.73 m^2^)	84.8 ± 14.4	41 ± 16.6	-	-	-	-
VD SVP—IR	0.36 ± 0.03	0.34 ± 0.04	0.033	0.265	0.01	0.051
IS	0.37 ± 0.04	0.35 ± 0.05	0.255 *	-	0.082 *	-
IT	0.36 ± 0.04	0.34 ± 0.04	0.362 *	-	0.135 *	-
II	0.37 ± 0.04	0.35 ± 0.04	0.099 *	-	0.04 *	0.448
IN	0.36 ± 0.03	0.34 ± 0.05	0.19 *	-	0.1 *	-
VD SVP—OR	0.35 ± 0.02	0.33 ± 0.04	0.017	0.145	0.08	0.066
OS	0.35 ± 0.03	0.33 ± 0.05	0.074 *	-	0.087 *	-
OT	0.32 ± 0.04	0.29 ± 0.04	0.199 *	-	0.216 *	-
OI	0.34 ± 0.03	0.33 ± 0.04	0.243 *	-	0.207 *	-
ON	0.39 ± 0.02	0.38 ± 0.04	0.065 *	-	0.088 *	-
VD DVP—IR	0.24 ± 0.03	0.23 ± 0.03	0.4	-	0.28	-
IS	0.23 ± 0.03	0.23 ± 0.03	1 *	-	1 *	-
IT	0.23 ± 0.04	0.22 ± 0.04	1 *	-	1 *	-
II	0.24 ± 0.02	0.23 ± 0.03	0.293 *	-	0.145 *	-
IN	0.24 ± 0.04	0.23 ± 0.03	1 *	-	0.893 *	-
VD DVP—OR	0.24 ± 0.01	0.23 ± 0.02	0.003	0.261	0.001	0.163
OS	0.23 ± 0.02	0.22 ± 0.03	0.061 *	-	0.026 *	0.132
OT	0.25 ± 0.03	0.24 ± 0.03	0.417 *	-	0.35 *	-
OI	0.23 ± 0.02	0.23 ± 0.02	1 *	-	0.522 *	-
ON	0.26 ± 0.03	0.24 ± 0.02	0.038 *	0.634	0.044 *	0.373
FD SVP	1.87 ± 0.01	1.86 ± 0.02	0.024	0.143	0.018	0.054
FD DVP	1.83 ± 0.01	1.82 ± 0.01	0.006	0.378	0.002	0.331
FAZ SVP	247 ± 86.8	209 ± 73.9	0.048	0.105	0.283	0.119

CKD = chronic kidney disease, DVP = deep vascular plexus, FAZ = foveal avascular zone, FD = fractal dimension, GFR = glomerular filtration rate, II = inner inferior, IN = inner nasal, IR = inner ring, IS = inner superior, IT = inner temporal, OI = outer inferior, ON = outer nasal, OR = outer ring, OS = outer superior, OT = outer temporal, SD = standard deviation, SVP = superficial vascular plexus, VD = vessel density. * Bonferroni correction was applied to adjust for the number of subareas.

## Data Availability

Data available upon reasonable request.

## References

[1] National Kidney Foundation (2002). K/DOQI Clinical Practice Guidelines for chronic Kidney Disease: Evaluation, Classification, and Stratification. Am. J. Kidney Dis..

[2] Kang D.H., Kanellis J., Hugo C., Truong L., Anderson S., Kerjaschki D., Schreiner G.F., Johnson R.J. (2002). Role of the microvascular endothelium in progressive renal disease. J. Am. Soc. Nephrol..

[3] Ooi Q.L., Newk-Fon Hey Tow F.K., Deva R., Alias M.A., Kawasaki R., Wong T.Y., Mohamad N., Colville D., Hutchinson A., Savige J. (2011). The microvasculature in chronic kidney disease. Clin. J. Am. Soc. Nephrol..

[4] Paterson E.N., Ravindran M.L., Griffiths K., Le Velly C.A., Cardwell C.C., McCarter R.V., Nicol P., Chhablani J.K., Rasheed M.A., Vupparaboina K.K. (2020). Association of reduced inner retinal thicknesses with chronic kidney disease. BMC Nephrol..

[5] Sabanayagam C., Shankar A., Klein B.E.K., Lee K.E., Muntner P., Nieto F.J., Tsai M.Y., Cruickshanks K.J., Schubert C.R., Brazy P.C. (2011). Bidirectional association of retinal vessel diameters and estimated GFR decline: The beaver dam CKD study. Am. J. Kidney Dis..

[6] Sabanayagam C., Xu D., Ting D.S.W., Nusinovici S., Banu R., Hamzah H., Lim C., Tham Y.C., Cheung C.Y., Tai E.S. (2020). A deep learning algorithm to detect chronic kidney disease from retinal photographs in community-based populations. Lancet Digit. Health.

[7] Sng C.C.A., Sabanayagam C., Lamoureux E.L., Liu E., Lim S.C., Hamzah H., Lee J., Tai E.S., Wong T.Y. (2010). Fractal analysis of the retinal vasculature and chronic kidney disease. Nephrol. Dial. Transplant..

[8] Yau J.W.Y., Xie J., Kawasaki R., Kramer H., Shlipak M., Klein R., Klein B., Cotch M.F., Wong T.Y. (2011). Retinal arteriolar narrowing and subsequent development of CKD stage 3: The multi-ethnic study of atherosclerosis (MESA). Am. J. Kidney Dis..

[9] Sabanayagam C., Tai E.S., Shankar A., Lee J., Sun C., Wong T.Y. (2009). Retinal arteriolar narrowing increases the likelihood of chronic kidney disease in hypertension. J. Hypertens..

[10] Liew G., Wang J.J., Mitchell P., Wong T.Y. (2008). Retinal vascular imaging: A new tool in microvascular disease research. Circ. Cardiovasc. Imaging.

[11] Wong C.W., Wong T.Y., Cheng C.Y., Sabanayagam C. (2014). Kidney and eye diseases: Common risk factors, etiological mechanisms, and pathways. Kidney Int..

[12] Tien Y.W., Islam F.M.A., Klein R., Klein B.E.K., Cotch M.F., Castro C., Sharrett A.R., Shahar E. (2006). Retinal vascular caliber, cardiovascular risk factors, and inflammation: The Multi-Ethnic Study of Atherosclerosis (MESA). Investig. Ophthalmol. Vis. Sci..

[13] Ikram M.K., De Jong F.J., Vingerling J.R., Witteman J.C.M., Hofman A., Breteler M.M.B., De Jong P.T.V.M. (2004). Are retinal arteriolar or venular diameters associated with markers for cardiovascular disorders? The Rotterdam study. Investig. Ophthalmol. Vis. Sci..

[14] Liew G., Sharrett A.R., Wang J.J., Klein R., Klein B.E.K., Mitchell P., Wong T.Y. (2008). Relative importance of systemic determinants of retinal arteriolar and venular caliber: The atherosclerosis risk in communities study. Arch. Ophthalmol..

[15] Islam F.M.A., Nguyen T.T., Wang J.J., Tai E.S., Shankar A., Saw S.M., Aung T., Lim S.C., Mitchell P., Wong T.Y. (2009). Quantitative retinal vascular calibre changes in diabetes and retinopathy: The Singapore Malay eye study. Eye.

[16] Lim L.S., Cheung C.Y.L., Sabanayagam C., Lim S.C., Tai E.S., Huang L., Wong T.Y. (2013). Structural changes in the retinal microvasculature and renal function. Investig. Ophthalmol. Vis. Sci..

[17] Broe R., Rasmussen M.L., Frydkjaer-Olsen U., Olsen B.S., Mortensen H.B., Peto T., Grauslund J. (2014). Retinal vascular fractals predict long-term microvascular complications in type 1 diabetes mellitus: The Danish Cohort of Pediatric Diabetes 1987 (DCPD1987). Diabetologia.

[18] Wu I.W., Sun C.C., Lee C.C., Liu C.F., Wong T.Y., Chen S.Y., Huang J.C.C., Tseng C.H., Yeung L. (2020). Retinal neurovascular changes in chronic kidney disease. Acta Ophthalmol..

[19] Wang W., He M., Gong X., Wang L., Meng J., Li Y., Xiong K., Li W., Huang W. (2020). Association of renal function with retinal vessel density in patients with type 2 diabetes by using swept-source optical coherence tomographic angiography. Br. J. Ophthalmol..

[20] Peng Q., Hu Y., Huang M., Wu Y., Zhong P., Dong X., Wu Q., Liu B., Li C., Xie J. (2020). Retinal neurovascular impairment in patients with essential hypertension: An optical coherence tomography angiography study. Investig. Ophthalmol. Vis. Sci..

[21] Inker L.A., Schmid C.H., Tighiouart H., Eckfeldt J.H., Feldman H.I., Greene T., Kusek J.W., Manzi J., Van Lente F., Zhang Y.L. (2012). Estimating Glomerular Filtration Rate from Serum Creatinine and Cystatin C. N. Engl. J. Med..

[22] Yu S., Lakshminarayanan V. (2021). Fractal dimension and retinal pathology: A meta-analysis. Appl. Sci..

[23] Ong Y.T., Wong T.Y., Klein R., Klein B.E.K., Mitchell P., Sharrett A.R., Couper D.J., Ikram M.K. (2013). Hypertensive retinopathy and risk of stroke. Hypertension.

[24] Grunwald J.E., Alexander J., Ying G.S., Maguire M., Daniel E., Whittock-Martin R., Parker C., McWilliams K., Lo J.C., Go A. (2012). Retinopathy and chronic kidney disease in the Chronic Renal Insufficiency Cohort (CRIC) study. Arch. Ophthalmol..

[25] Wong T.Y., Coresh J., Klein R., Muntner P., Couper D.J., Sharrett A.R., Klein B.E.K., Heiss G., Hubbard L.D., Duncan B.B. (2004). Retinal microvascular abnormalities and renal dysfunction: The Atherosclerosis Risk in Communities Study. J. Am. Soc. Nephrol..

[26] Wang Q., Liu L., Jonas J.B., Gao B., Wu S.L., Chen S.H., Yan Y.N., Yang J.Y., Zhou W.J., Yang M.C. (2021). Albuminuria and retinal vessel density in diabetes without diabetic retinopathy: The Kailuan Eye Study. Acta Ophthalmol..

[27] You Q.S., Chan J.C.H., Ng A.L.K., Choy B.K.N., Shih K.C., Cheung J.J.C., Wong J.K.W., Shum J.W.H., Ni M.Y., Lai J.S.M. (2019). Macular Vessel Density Measured With Optical Coherence Tomography Angiography and Its Associations in a Large Population-Based Study. Investig. Ophthalmol. Vis. Sci..

[28] Hua D., Xu Y., Zeng X., Yang N., Jiang M., Zhang X., Yang J., He T., Xing Y. (2020). Use of optical coherence tomography angiography for assessment of microvascular changes in the macula and optic nerve head in hypertensive patients without hypertensive retinopathy. Microvasc. Res..

[29] Hua D., Xu Y., Zhang X., He T., Chen C., Chen Z., Xing Y. (2021). Retinal Microvascular Changes in Hypertensive Patients with Different Levels of Blood Pressure Control and without Hypertensive Retinopathy. Curr. Eye Res..

[30] Liu Y., Li J., Pan J., Wang Y., Mao G., Jiang Z. (2021). Morphological changes in and quantitative analysis of macular retinal microvasculature by optical coherence tomography angiography in hypertensive retinopathy. Hypertens. Res..

[31] Mainster M.A. (1990). The fractal properties of retinal vessels: Embryological and clinical implications. Eye.

[32] Zahid S., Dolz-Marco R., Freund K.B., Balaratnasingam C., Dansingani K., Gilani F., Mehta N., Young E., Klifto M.R., Chae B. (2016). Fractal dimensional analysis of optical coherence tomography angiography in eyes with diabetic retinopathy. Investig. Ophthalmol. Vis. Sci..

[33] Lin Y., Jiang H., Liu Y., Gameiro G.R., Gregori G., Dong C., Rundek T., Wang J. (2019). Age-related alterations in retinal tissue perfusion and volumetric vessel density. Investig. Ophthalmol. Vis. Sci..

[34] Jo Y.H., Sung K.R., Shin J.W. (2019). Effects of age on peripapillary and macular vessel density determined using optical coherence tomography angiography in healthy eyes. Investig. Ophthalmol. Vis. Sci..

[35] O’Hare A.M., Choi A.I., Bertenthal D., Bacchetti P., Garg A.X., Kaufman J.S., Walter L.C., Mehta K.M., Steinman M.A., Allon M. (2007). Age affects outcomes in chronic kidney disease. J. Am. Soc. Nephrol..

